# The effects of pregnancy discrimination on postpartum depressive symptoms: a follow-up study

**DOI:** 10.1186/s12884-022-05148-2

**Published:** 2022-11-08

**Authors:** Yuko Kachi, Takeo Fujiwara, Akiomi Inoue, Sachiko Baba, Hisashi Eguchi, Hiroshi Ohta, Akizumi Tsutsumi

**Affiliations:** 1 Department of Public Health, Kitasato University School of Medicine, 1-15-1 Kitasato, Minami-Ku, Sagamihara, Kanagawa 252-0374 Japan; 2 Department of Global Health Promotion, Tokyo Medical and Dental University, 1-5-45 Yushima, Bunkyo-Ku, Tokyo, 113-8510 Japan; 3 Institutional Research Center, University of Occupational and Environmental Health, Japan, 1-1 Iseigaoka, Yahatanishi-Ku, Kitakyushu, 807-8555 Japan; 4 Division of Community Health and Research, Maternal and Child Health Information Center, Osaka Women’s and Children’s Hospital, 840 Murodo-Cho, Izumi, Osaka 594-1101 Japan; 5 Department of Mental Health, Institute of Industrial Ecological Sciences, University of Occupational and Environmental Health, Japan, 1-1 Iseigaoka, Yahatanishi-Ku, Kitakyushu, 807-8555 Japan; 6 Matsugishi Ladies’ Clinic, 3-43-1 Kozunomori, Narita, Chiba 286-0048 Japan

**Keywords:** Pregnancy discrimination, Postpartum depression, Precarious employment, Follow-up study, Mediation analysis

## Abstract

**Background:**

Pregnancy discrimination in the workplace is prevalent worldwide. However, few studies have examined the effects of pregnancy discrimination on mothers’ perinatal mental health. We aimed to investigate the association between pregnancy discrimination and postpartum depressive symptoms, and the mediation effects of prenatal depressive symptoms on this association.

**Methods:**

Our sample consisted of 285 Japanese women employed during pregnancy who completed a baseline online survey in May 2020 and a follow-up mail survey two months postpartum. Pregnancy discrimination was defined as exposure to any of 16 forms of disadvantageous treatment or harassment related to pregnancy, prohibited by national guidelines. Prenatal (assessed at baseline) and postpartum (assessed at follow-up) depressive symptoms were measured using the Edinburgh Postnatal Depression Scale. Multiple linear regression and mediation analyses were performed overall and stratified by regular (permanent) and non-regular (precarious) employees.

**Results:**

Overall, 23.9% of participants experienced pregnancy discrimination during pregnancy. After adjusting for potential confounders, pregnancy discrimination was significantly associated with postpartum depressive symptoms (coefficient 1.76, 95% confidence interval [CI] 0.65–2.88). When stratified by employment type, these effects were observable among non-regular employees (coefficient 2.51, 95% CI 0.45–4.57) but not regular employees. Mediation analysis showed that prenatal depressive symptoms mediated 57.1% (95% CI 20.1–94.1%) of the association between pregnancy discrimination and postpartum depressive symptoms among all participants, with a greater effect among non-regular employees (64.1% [95% CI 18.5–109.8%]).

**Conclusions:**

Pregnancy discrimination has adverse effects on postpartum depressive symptoms, partially through prenatal depressive symptoms, especially among non-regular employees. To prevent perinatal depression in female workers, employers should comply with legislation and take preventive measures against pregnancy discrimination, while considering vulnerable employees.

**Supplementary Information:**

The online version contains supplementary material available at 10.1186/s12884-022-05148-2.

## Background

Pregnancy discrimination is a serious global problem, even in countries where legal protections are in place [1]. In a UK survey, three out of four mothers reported having negative or possibly discriminatory experiences related to pregnancy, some of which did not fall within legal definitions of discrimination [2]. Pregnancy discrimination is defined by Japanese law as disadvantageous treatment or harassment of women in the workplace due to pregnancy, childbirth, and requesting or taking childcare/family care leave, among others [3]. In Japan, one in four to five women had experienced pregnancy discrimination, but about half did not disclose their experiences to anyone or seek resolution [4–6]. In Japan, pregnancy discrimination is prohibited by the Equal Employment Opportunity Act (amended in 2007) and the Child Care and Family Care Leave Act (amended in 2001), requiring employers to take measures to prevent pregnancy discrimination; however, there is no provision for penalties in cases of non-compliance [3]. In addition, few women file lawsuits for pregnancy discrimination in Japan [7] because of the low compensation, in contrast to the approximately 40,000 pregnancy discrimination lawsuits filed in the United States (US) over the past decade [8].

Workplace bullying adversely impacts mental health [9, 10]. Pregnancy discrimination is a form of workplace bullying that occurs during periods of mental health vulnerability due to pregnancy and childcare, with possible serious effects on perinatal mental health. In addition, prenatal and postpartum maternal depressive symptoms or depression increase the risk of negative outcomes, such as premature delivery, impaired mother–child bonding, and infant death and hospitalization in the first year of life [11–14]. However, only a few studies have investigated the effects of pregnancy discrimination on mental health worldwide [15].

A US study prospectively examined the effects of pregnancy discrimination on mothers’ postpartum depressive symptoms and infants’ health using online surveys, distributed to 252 pregnant women employed full-time. They found that perceived pregnancy discrimination was indirectly associated with postpartum depressive symptoms in mothers, and low birth weight and low gestational age in babies, via mothers’ perceived stress during pregnancy [15]. In addition, a Japanese cross-sectional study of 255 working pregnant women attending obstetric care facilities found that pregnancy discrimination was associated with depression and negative feelings towards the fetus [16]. However, as noted above, there has been only one longitudinal study thus far [15], limiting generalizability. Moreover, workers in vulnerable positions in the workplace, i.e., non-regular employees, who are employed on a fixed-term or indirect basis (e.g., part-time, contract, or temporary employees), may be more susceptible to workplace bullying than regular workers who are employed indefinitely and directly. Non-regular workers tend to be subjected to a variety of unfavorable working conditions, including low income, job insecurity, lack of job training, and unfair treatment, such as bullying [17]. However, no differences in the association between pregnancy discrimination and mental health between regular and non-regular employees have been examined.

Our cross-sectional survey of 359 pregnant employees during the coronavirus disease 2019 (COVID-19) pandemic in 2020 [5] showed that 25% of pregnant employees had experienced pregnancy discrimination and had a higher prevalence of depression than those who had not experienced pregnancy discrimination. COVID-19-related business closures worsened the management of the service sector and rendered non-regular employees unemployable [18], which may also have disproportionally increased pregnancy discrimination among non-regular employees compared to regular employees. Thereafter, we followed up on the depressive symptoms of the same women two months postpartum.

The current study aimed to prospectively examine the effects of pregnancy discrimination on postpartum depressive symptoms among women who had been employed during pregnancy, overall and by employment status (i.e., regular vs. non-regular employment). As prenatal depression is a predictor of postpartum depression [19], we also examined whether prenatal depressive symptoms mediate the association between pregnancy discrimination and postpartum depressive symptoms. In our analyses, we adjusted for pregnant women's fear of infection and changes in work arrangements, such as telecommuting, that occurred due to the COVID-19 pandemic, because major changes in life and threats to maternal and child health can be associated with perinatal depression [20].

## Methods

### Participants

This was a follow-up study using a cohort of pregnant women described in a previous publication [5]. The baseline survey was conducted online, during pregnancy, between May 22 and 31, 2020, and the follow-up survey was conducted by mail at two months postpartum. The survey was conducted by the Japan Management Association (JMA) Research Institute Inc., an online survey management company based in Japan. From their online survey registrants, the baseline survey recruited 400 working pregnant women after eight weeks of pregnancy (including women who had already left the workforce at the time of the survey, but who were working at the time their pregnancy was confirmed). JMA Research Institute Inc. called these participants two months after their due date to confirm that they had given birth and mailed self-administered questionnaires to the home addresses of participants who had given birth. The follow-up survey was conducted by mail rather than online as some participants might have unregistered the online survey during follow-up. A total of 318 women returned the questionnaire (follow-up rate: 79.5%).

We restricted our analyses to women employed during pregnancy and excluded eight corporate officers, six self-employed women, and four with unknown employment status. We also excluded 15 women with a history of mental disorders. A total of 285 women were included in this study. Descriptively comparing the characteristics of those who remained in the study versus those who dropped out shows that the latter were more likely to be junior high or high school graduates, experience fear of COVID-19 more often, be non-regular workers, work nights or shifts, and have higher depressive symptom scores at baseline.

This study was approved by Kitasato University Medical Ethics Organization (No. B18-281). Prior to participating in the online survey, the respondents read an explanation of the survey and understood that participation was voluntary. Receipt of the completed surveys was assumed to indicate consent to participate. JMA Research Inc. provided the data in an anonymized format; individual respondents could not be identified. Participants who responded to both the baseline and follow-up surveys received small monetary awards.

### Postpartum depressive symptoms

Postpartum depressive symptoms were measured in a follow-up survey using the Japanese version of the Edinburgh Postnatal Depression Scale (EPDS). This 10-item self-rated scale has been widely used to screen for prenatal and postpartum depression [21, 22]. The scale rates the intensity of depressive symptoms within the previous seven days. Each item is scored on a four-point Likert scale (ranging from 0 to 3), and the total score ranges from 0 to 30. Cronbach’s α coefficient for the current sample was 0.84.

### Pregnancy discrimination

Pregnancy discrimination was measured during the baseline survey. Following a previous study (JILPT 2016), pregnancy discrimination was assessed by asking respondents whether they had ever experienced any of the 16 forms of disadvantageous treatment or harassment by supervisors and/or colleagues (e.g., dismissal, pay cut, and physical or mental harassment; see Supplemental Table 1), which are listed as specific examples of disadvantageous treatment prohibited by the national guidelines [23]. Those who had left their jobs were asked about their experiences at work after their pregnancies had been confirmed. Respondents who had experienced one or more of the 16 items were classified as having experienced pregnancy discrimination.

### Potential confounders

Potential confounders were measured in the baseline survey and included age (continuous), education (junior high/high school or junior/4-year college or greater), number of weeks pregnant (first trimester [i.e., 8–13 weeks], second trimester [i.e., 14–27 weeks], and third trimester [i.e., 28–41 weeks]), type of employment (regular or non-regular), company size (1–299 employees, 300 or more employees, civil service office), occupation (manager/professional/technician, clerical/sales/service, security/transportation/labor, and others), weekly working hours (≤ 39, 40–49, and ≥ 50 h), work schedule (day shift only or night/shift work), work mode during the COVID-19 pandemic (telecommuting, working in an office, or retired), and frequency of fear of COVID-19 (low or high).

Type of employment was assessed by a question on employment status with four response options and classified into two categories: regular employment (full-time permanent employees) and non-regular employment (part-time employees, contract or entrusted employees, or employees from temporary labor agencies). Company size was classified based on the definition of small- and medium-sized companies listed in the Japan Small- and Medium-sized Enterprise Basic Act. Civil service offices were classified into a separate category, regardless of size. The frequency of fear of COVID-19 was assessed by the question, “Did you worry or fear infection with the novel coronavirus?” Responses were divided into two categories: low (not at all or sometimes) or high (often or always).

### Mediator

Prenatal depressive symptoms were treated together as a mediator and measured in the baseline survey using the Japanese version of the EPDS. Cronbach’s α coefficient for the current sample was 0.87.

### Statistical analysis

**Fig. 1:**
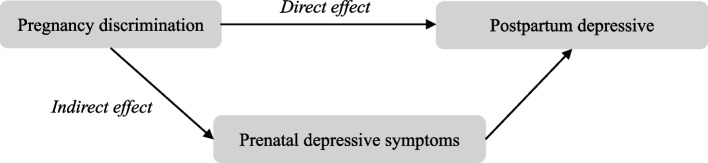
Conceptual diagram

We first described respondents’ characteristics as either percentages or means (standard deviation [SD]), based on whether they had experienced pregnancy discrimination. Next, a multiple linear regression analysis was performed with postpartum depressive symptoms (continous) as a dependent variable and pregnancy discrimination (binary) as an independent variable. We estimated a crude and adjusted model, including all confounders. In addition, we conducted stratified analysis by type of employment to examine whether the effects of pregnancy discrimination on postpartum depressive symptoms differed between regular and non-regular employees. The multiple regression analyses confirmed the basic assumption that the residuals are normally distributed. Additionally, no interactions were found (*p* > 0.05) when we tested the interaction between pregnancy discrimination and type of employment prior to modeling. Finally, we conducted a mediation analysis using the method proposed by Lange et al. [24] to assess the effect mediated by prenatal depressive symptoms in the potential causal pathway between pregnancy discrimination and postpartum depressive symptoms. Their method relies on a counterfactual framework approximated by inverse probability weighting, which enabled our study’s analysis to decompose the total effect of pregnancy discrimination on postpartum depression into 1) a direct effect of pregnancy discrimination and 2) an indirect effect of pregnancy discrimination mediated through prenatal depressive symptoms, after adjusting for all confounders (Fig. 1). All statistical tests were two-sided with a significance level of 5%. All analyses were performed using SAS version 9.4 for Windows (SAS Inc., Cary, NC, USA).

## Results

Overall, 23.9% of the participants experienced pregnancy discrimination during pregnancy (Supplemental Table 1). Table 1 shows participants’ characteristics based on their pregnancy discrimination experiences. Participants who experienced pregnancy discrimination were more likely to have the following characteristics compared to those who did not experience pregnancy discrimination: lower education levels, smaller companies, working fewer hours per week, and high frequency of fear of COVID-19 infection. The postpartum depressive symptoms score (mean ± SD) was higher in participants who experienced pregnancy discrimination than those who did not (5.7 ± 3.9 vs. 4.0 ± 3.8; *p* = 0.003).

**Table 1:** Characteristics of participants with experience of pregnancy discrimination during pregnancy (*N* = 285)

	Experience	No experience
	(*n* = 68)	(*n* = 217)
	N(%) or mean ± SD	
***Demographics and pregnancy status***		
Age		
mean ± SD years	31.5 ± 4.6	31.5 ± 4.3
Education		
Junior high/ high school	11 (16.2)	21 (9.7)
Junior /4-year college or greater	57 (83.8)	196 (90.3)
Number of weeks pregnant		
First trimester	6 (8.8)	22 (10.1)
Second trimester	27 (39.7)	87 (40.1)
Third trimester	35 (51.5)	108 (49.8)
***Work Status***		
Type of employment		
Regular	44 (64.7)	141 (65.0)
Non-regular	24 (35.3)	76 (35.0)
Company size		
1–299 employees	37 (54.4)	86 (39.6)
300 or more employees	26 (38.2)	127 (58.5)
Civil service office	5 (7.4)	4 (1.8)
Occupation		
Manager/professional/technician	19 (27.9)	71 (32.7)
Clerical/sales/service	45 (66.2)	125 (57.6)
Security/transportation/labor	3 (4.4)	4 (1.8)
Others	1 (1.5)	17 (7.8)
Weekly working hours		
≤ 39 h	39 (57.4)	103 (47.5)
40–49 h	23 (33.8)	103 (47.5)
≥ 50 h	6 (8.8)	11 (5.1)
Work schedule		
Dayshift only	62 (91.2)	204 (94.0)
Night/shift work	6 (8.8)	13 (6.0)
***COVID-19***		
Work mode		
Telecommuting	27 (39.7)	95 (43.8)
Working in an office	32 (47.1)	97 (44.7)
Retired	9 (13.2)	25 (11.5)
Frequency of fear of COVID-19		
Low	28 (41.2)	123 (56.7)
BHigh	40 (58.8)	94 (43.3)
*** Depressive symptoms***^**a**^		
Prenatal	9.0 ± 6.0	5.6 ± 4.5
Postpartum	5.7 ± 3.9	4.0 ± 3.8

^a^ mean Edinburgh postnatal depression scale score

The results of a multiple regression analysis (Table 2) show that, overall, pregnancy discrimination was significantly associated with postpartum depressive symptoms both in the crude (B = 1.66, 95% confidence interval [CI]: 0.61–2.71) and adjusted models (B = 1.76, 95% CI: 0.65–2.88). When stratified by type of employment, we observed a significant association between pregnancy discrimination and postpartum depressive symptoms for regular employees in the crude (B = 1.33, 95% CI: 0.01–2.65) but not in the adjusted analyses. For non-regular employees, a significant association was observed in both the crude (B = 2.27, 95% CI: 0.49–4.04) and adjusted models (B = 2.51, 95% CI: 0.45–4.57).

**Table 2:** The effects of pregnancy discrimination during pregnancy on postpartum depressive symptoms, overall and stratified by type of employment (*N* = 285)

	Crude model		Adjusted model^a^	
Pregnancy discrimination	*B*	(95%CI)	*B*	(95%CI)
Overall (*N* = 285)				
Experience	1.66	(0.61, 2.71)^*^	1.76	(0.65, 2.88)^*^
No experience	Ref		Ref	
*R*^*2*^	0.03		0.09	
Stratified by type of employment				
Regular (*N* = 185)				
Experience	1.33	(0.01, 2.65)^*^	1.17	(-0.26, 2.60)
No experience	Ref		Ref	
*R*^*2*^	0.02		0.12	
Non-regular (*N* = 100)				
Experience	2.27	(0.49, 4.04)^*^	2.51	(0.45, 4.57)^*^
No experience	Ref		Ref	
*R*^*2*^	0.06		0.16	

*B* = unstandardized coefficient; 95%CI = 95% confidence interval

^*^p < 0.05

^a^Adjusted for age, education, number of weeks pregnant, type of employment (excluded in stratified analysis), company size, occupation, weekly working hours, work schedule, work mode, and frequency of fear of COVID-19

The results of the mediation analysis (Table 3) show that the total effect of pregnancy discrimination on postpartum depressive symptoms (B = 1.76, 95% CI: 0.68–2.84) in all participants was decomposed into a direct pregnancy discrimination effect of B = 0.75 (95% CI: -0.27–1.78) and an indirect pregnancy discrimination effect mediated through prenatal depressive symptoms of B = 1.01 (95% CI: 0.50–1.51). Similarly, the total effect (B = 2.51, 95% CI: 0.65–4.37) in non-regular employees was decomposed into a direct effect of B = 0.90 (95% CI: -0.69–2.50) and an indirect pregnancy discrimination effect mediated through prenatal depressive symptoms of B = 1.61 (95% CI: 0.46–2.76). Therefore, prenatal depressive symptoms mediated 57.1% (95% CI: 20.1–94.1%) of the association between pregnancy discrimination and postpartum depressive symptoms in all participants, and 64.1% (95% CI: 18.5–109.8%) of the associations in non-regular employees.

**Table 3:** Total, direct, and indirect effects of the associations between pregnancy discrimination during pregnancy and postpartum depressive symptoms, overall and stratified by type of employment

	Overall (*N* = 285)		Regular employees (*N* = 185)		Non-regular employees (*N* = 100)	
	*B*	(95%CI)	*B*	(95%CI)	*B*	(95%CI)
Total effect	1.76	(0.68, 2.84)^*^	1.17	(-0.18, 2.52)	2.51	(0.65, 4.37)^*^
Direct effect	0.75	(-0.27, 1.78)	0.54	(-0.79, 1.86)	0.90	(-0.69, 2.50)
Indirect effect	1.01	(0.50, 1.51)^*^	0.63	(0.13, 1.13)^*^	1.61	(0.46, 2.76)^*^
% mediated	57.1% (20.1%, 94.1%)		54.2% (-12.4%, 120.8%)		64.1% (18.5%, 109.8%)	

Adjusted for age, education, number of weeks pregnant, type of employment (excluded in stratified analysis), company size, occupation, weekly working hours, work schedule, work mode, and frequency of fear of COVID-19

*B* = unstandardized coefficient; 95%CI = 95% confidence interval

^*^p < 0.05

## Discussion

This prospective study demonstrated that pregnancy discrimination had adverse effects on postpartum depressive symptoms, partially mediated through prenatal depressive symptoms. This association was particularly pronounced among non-regular employees. Although our study was conducted under the unique circumstances of the COVID-19 pandemic, these associations persisted after adjusting for fear of COVID-19 and infection control measures in the workplace, such as teleworking.

We observed a significant indirect association between pregnancy discrimination and postpartum depressive symptoms, mediated by preterm depressive symptoms. This result is consistent with the findings of a US longitudinal study [15], suggesting that perceived pregnancy discrimination is indirectly associated with mothers’ postpartum depressive symptoms via perceived stress during pregnancy. In the US, pregnancy discrimination victims often file lawsuits [8], whereas in Japan, most victims do not file lawsuits and endure pregnancy discrimination instead [7]. The fact that similar results were obtained despite these differences in the cultural context suggests that strengthening penalties against pregnancy discrimination and facilitating litigation alone will not eliminate pregnancy discrimination or its negative effects on mental health. Prior research has suggested that traditional gender norms and inequality in the workplace are linked to pregnancy discrimination [25, 26]. In addition, pregnant women and mothers in the workplace are more likely to be stereotyped as emotional, irrational, less committed to their work, and less competent than other employees [27, 28]. Such stereotypes against women can also lead to pregnancy discrimination [29]. Therefore, the promotion of gender equality in the workplace and the elimination of stereotypes against women may be necessary to prevent pregnancy discrimination.

The effect of pregnancy discrimination on postpartum depressive symptoms was more pronounced in non-regular than regular workers. Most non-regular employment is characterized by poor job security, low wages, workplace hazards, and psychosocially stressful working conditions [17]. Consistent with this situation, our baseline study [5] showed that the experience rate of pregnancy discrimination was higher among non-regular than regular employees; however, in the current follow-up study, there was no difference in the experience rate of pregnancy discrimination because more non-regular employees dropped out during the follow-up survey than regular employees. In addition, non-regular workers are more likely to develop depression than regular workers due to the abovementioned unfavorable working conditions [30–32]. The pronounced association between pregnancy discrimination and postpartum depressive symptoms among non-regular workers is consistent with these findings. However, since this survey was conducted when the employment of non-regular workers was threatened due to COVID-19 related business downturn, it is possible that the association was noticeable and requires re-examination when the pandemic settles down.

Our mediation analysis partially revealed the mechanisms underlying the association between pregnancy discrimination and postpartum depressive symptoms. Pregnancy discrimination during pregnancy may cause prenatal depressive symptoms that persist postpartum. The association between pregnancy discrimination and prenatal depressive symptoms can be explained by the job stress model: exposure to pregnancy discrimination as a work-related stressor may lead to prenatal depressive symptoms as a stress response [33]. Moreover, prenatal depression is known to be on a continuum with postpartum depression [19]. An increasing number of studies have demonstrated associations between several work-related stressors and depression risk during prenatal and postnatal periods [34, 35].

The strengths of this study include its prospective design and high response rate. Furthermore, it is the first study in Japan to demonstrate an indirect association between pregnancy discrimination and postpartum depressive symptoms mediated by prenatal depressive symptoms. However, our study also has some limitations. First, the small sample size may have obscured any significant associations. Second, because the data for the predictor, mediator, and outcome variables were based on respondents’ self-reports, an underlying negative reporting style or negative mood could have led to a spurious association between pregnancy discrimination and postpartum depressive symptoms. However, the exclusion of those with a history of mental disorders and the adjustment for the frequency of fear of COVID-19 may partly decrease the possible confounding effects of negative reporting style or negative mood. Third, although the pregnancy discrimination measurement items in this study were based on national guidelines, they may not have had sufficient validity. Finally, the generalizability of our findings is limited because the study participants were recruited from online survey registrants held by contract research companies during the COVID-19 pandemic.

## Conclusion

The results of this follow-up study indicate that pregnancy discrimination is associated with postpartum depressive symptoms, partially through prenatal depressive symptoms. This association was more pronounced among non-regular employees. Future studies should replicate our findings using larger sample sizes and examine the protective factors of this association. To protect the health of female workers and their children before and after pregnancy, employers should not only comply with pregnancy discrimination legislation but also improve work environments to eliminate discrimination against women.

## Supplementary Information

**Additional file 1: Supplemental Table 1.** The experience of pregnancy discrimination at baseline (*N* = 285).

## Data Availability

The datasets presented in this article are not readily available because due to the nature of this research, participants of this study did not agree for their data to be shared publicly, so supporting data is not available. Requests to access the datasets should be directed to kachi@med.kitasato-u.ac.jp.
