# Supplementary material for: The effects of pregnancy discrimination on postpartum depressive symptoms: a follow-up study
Source: BMC Pregnancy Childbirth. 2022 Nov 8;22:825. doi: 10.1186/s12884-022-05148-2 (PMC9641687; doi:10.1186/s12884-022-05148-2)
Supplement: Supplementary file 1 — Additional file 1: Supplemental Table 1. The experience of pregnancy discrimination at baseline (N = 285). [file 12884_2022_5148_MOESM1_ESM.docx]

| **Supplemental Table 1.　The experience of pregnancy discrimination at baseline（N = 285）** | | | |
| --- | --- | --- | --- |
|  |  | N | (%) |
| 1 | Dismissal | 4 | (1.4) |
| 2 | The end of employment contract | 5 | (1.8) |
| 3 | Reduction in the number of contract renewals | 3 | (1.1) |
| 4 | Forcing you to resign | 9 | (3.2) |
| 5 | Forced conversion from permanent to non-permanent employees | 6 | (2.1) |
| 6 | Demotion | 4 | (1.4) |
| 7 | Pay cut | 10 | (3.5) |
| 8 | Unfavorable Calculation for Bonuses, etc. | 9 | (3.2) |
| 9 | Adverse reassignment | 11 | (3.9) |
| 10 | Unfavorable stay-at-home orders | 8 | (2.8) |
| 11 | Unfavorable evaluations in personnel evaluations for promotion and advancement | 8 | (2.8) |
| 12 | They wouldn't let me work, or they made me do exclusively menial tasks. | 9 | (3.2) |
| 13 | I've received statements that suggest one of 1-12. | 22 | (7.7) |
| 14 | I was prevented from using the systems related to pregnancy, childbirth, and childcare, such as maternity leave and childcare leave. | 14 | (4.9) |
| 15 | Mentally harassed (sarcastic, ignoring, etc.) | 28 | (9.8) |
| 16 | Physically harassed (forcing you to stand, smoking nearby, etc.) | 10 | (3.5) |
|  | One or more of the above 16 items | 68 | (23.9) |
